# Face Adaptation and Face Priming as Tools for Getting Insights Into the Quality of Face Space

**DOI:** 10.3389/fpsyg.2020.00166

**Published:** 2020-02-07

**Authors:** Ronja Mueller, Sandra Utz, Claus-Christian Carbon, Tilo Strobach

**Affiliations:** ^1^Medical School Hamburg, Hamburg, Germany; ^2^Bamberg Graduate School of Affective and Cognitive Sciences, University of Bamberg, Bamberg, Germany; ^3^Department of General Psychology and Methodology, University of Bamberg, Bamberg, Germany; ^4^Research Group EPAEG (Ergonomics, Psychological Æsthetics, Gestalt), Bamberg, Germany

**Keywords:** face space, face adaptation, face priming, mental representation, face perception, face processing model

## Abstract

During the recognition of faces, the incoming perceptual information is matched against mental representations of familiar faces stored in memory. Face space models describe an abstract concept of face representations and their mental organization, in which facial representations are located on various characteristic dimensions, depending on their specific facial characteristics. However, these models are defined just as incompletely as the general understanding of face recognition. We took two phenomena from face processing to better understand face recognition, and so the nature of face space: face adaptation and face priming. The face literature has mainly focused on face adaptation, largely neglecting face priming when trying to integrate outcomes regarding face recognition into the face space framework. Consequently, the present paper aims to review both phenomena and their contributions to face recognition, representation, and face space.

## Introduction

It is a common assumption that familiar faces are encoded and recognized by matching the incoming perceptual information against facial representations stored in memory (Bruce and Young, 1986). To discriminate faces from each other, these stored representations must contain a large variety of characteristics, continuously differing from each other along many dimensions (Lee et al., 2000). The ‘face space,’ according to Valentine (1991), describes an abstract concept, that considers these dimensional relationships among mental representations. Valentine proposes a multidimensional space, in which each facial representation is located, depending on its characteristic value on each dimension. According to this model, face representations that are located close to each other are similar, whereas representations that are further apart, share fewer similarities. The facial information these dimensions contain and on which the representations vary is not further specified. They could be global properties (e.g., age, gender, or ethnicity) or more specific facial parameters, such as the eye–mouth distance or the size of the head (Valentine et al., 2016). How many dimensions are needed to encode all human faces one encounters, is not known exactly. Through computational modeling, Lewis (2004) was able to narrow down the number of dimensions to between 15 and 22 (other authors, however, estimate the number of dimensions to be much higher; see, Meytlis and Sirovich, 2007).

**FIGURE 1:**
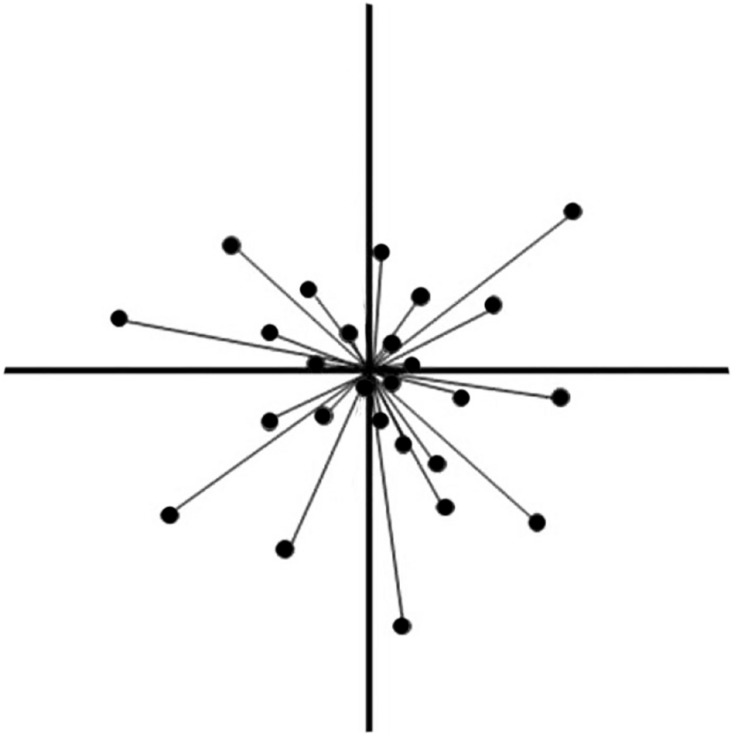
A two-dimensional illustration of the norm-based model of the face space proposed by Valentine (1991). The two dimensions are used for illustrative purpose only. The actual model contains multiple dimensions, on which faces can be distinguished. The points in the illustration represent the mental representations of familiar faces, which are located depending on their expressions on each dimension. The center represents the general face norm. The graph is based on the ideas of Valentine (1991). The permission and figure license has been obtained from the copyright holder [© SAGE Publications].

Within the face space framework several models about its specific structure exist. The two most well-known versions of the face space, the norm-based face space and the exemplar model, mainly differ in how the faces are arranged in space and in relation to each other (Valentine, 2001; Lewis, 2004; Valentine et al., 2016). The norm-based model assumes that faces are encoded relative to a specific prototypical face or central-norm face. Thus, a face must be located within the face space in relation to the norm. The deviation from the norm can be seen as a vector, in which the direction and the magnitude represent the distinctiveness and identity of a face (Figure 1). Within the exemplar model, however, faces are located in the face space without any reference to a norm or prototype. The distances between the face representations define the level of similarity (faces that are closer to each other are more similar; regarding facial similarity see also Tredoux, 2002). The distribution of the representations provide information about the distinctiveness (very distinct exemplars are located in areas of low representation density; Lewis, 2004; Valentine et al., 2016).

Studies investigating the perception, processing, and storage of faces can provide essential information about the functioning and structure of the face space. Two paradigms that are frequently used in this context are face adaptation and priming. In experimental settings, face adaptation effects are usually assessed by presenting familiar faces that were initially inspected as manipulated versions. In a subsequent test phase, participants are then asked to determine the veridical face out of the original and slightly altered faces. Results typically show a bias of the participants’ selection toward the previously inspected, manipulated version. This implies that the original face seems to be perceived as altered in the direction opposite to the adaptor face (Strobach and Carbon, 2013). Unlike basic, low-level adaptation effects on, e.g., color, motion or orientation, face adaptation effects seem to be very robust over time and thus suggest a high-level processing and an adaptation on a representational memory basis (Carbon and Ditye, 2011; Walther et al., 2013).

Priming is another phenomenon often used to demonstrate how recent perceptual experiences can alter the perception and recognition of faces. While adaptation usually leads to a perceptual bias opposite to the adaptor (i.e., original face versions are shifted away from the adaptation faces), priming often results in faster and/or more accurate responses in facial recognition after inspecting the same or similar faces (Ellis et al., 1987; Walther et al., 2013). Different priming paradigms can be distinguished, each addressing different mental concepts. For instance, repetition priming describes a paradigm in which a stimulus is initially presented as a prime and presented again in the subsequent test phase next to alternative stimuli. Repetition priming can facilitate the processing of that initially presented stimulus through activating its specific mental representation. Thus, the presentation of a face can facilitate the subsequent perception and recognition of the same face by activating its mental face representation (Ellis et al., 1993; Schweinberger et al., 1995). Semantic or associative priming characterizes a paradigm in which a stimulus is initially presented and an associated or unrelated stimulus is shown in the subsequent test phase. Associate priming can facilitate the processing of semantically related stimuli through activating a semantic network or an associated concept (Schweinberger et al., 1995; McNamara, 2005). Thus, the presentation of a person’s face can facilitate the recognition of an associated person (e.g., Barack and Michelle Obama) by activating a semantic network.

Both phenomena (i.e., face adaptation and priming) seem to differ substantially from each other and even seem to cause opposite effects (priming usually leads to an improvement of recognition and identification, whereas a correct facial identification becomes more difficult through adaptation). Nevertheless, they generally lead to a similar result: in one way or another, they both alter subsequent face recognition by either activating or altering mental representations of faces. Thus, both phenomena could potentially be used to gain insights into the structure and the characteristics of the face space. However, previous studies have so far mainly focused on adaptation when trying to integrate outcomes regarding face perception into the face space framework. Although priming could contribute as well to the understanding of the face space, this phenomenon has been mostly neglected in the face space literature. Consequently, the present paper aims to review both phenomena and their contributions to facial perception, processing and storage and thus also to the face space. To enable a systematic evaluation and categorization of the face adaptation and priming literature, both phenomena will be examined by taking into account two different dimensions: temporal aspects of the paradigms *(timing)* and the transferability of the effects to other face images or identities (*transfer*; Strobach and Carbon, 2013).

## Temporal Characteristics

The dimension *timing* categorizes adaptation and priming effects according to different temporal information. The first temporal information type *adaptor/prime duration* focuses on the length of the presentation time of the adaptor or prime. It reveals the impact the presentation duration can have on the adaptability or priming ability of faces. The second type of temporal information focuses on the *test duration*, the time span of the presented test stimuli. Similar to the *adaptor/prime duration*, this temporal information type provides insights into how the duration of the presentation of test faces can modulate the size of the adaptation or priming effects. Finally, we focus on the *delay*, the time interval between the adaptation or priming stimuli and the test phase. Here we distinguish between two general test designs: (1) a design in which a test stimulus is presented trial-wise after an adaptation or priming stimulus; and (2) a block-wise design in which there is first an adaptation or priming phase with several trials and then a separate test phase. The temporal information type *delay* provides essential information about the robustness and sustainability of adaptation or priming effects. It also gives us information about the recalibration ability of the visual system (meaning a recalibration back to the previous state of the visual system; Carbon and Ditye, 2011; Strobach and Carbon, 2013). The different temporal characteristics of all studies reported in this chapter, are summarized in Table 1.

**TABLE 1:** Adaptation/prime duration, delay and test duration in the selected face adaptation and priming studies, reported in this paper.

**Phenomenon**	**Study**	**Adaptor/prime duration**	**Delay**	**Test duration**
Adaptation	Carbon and Ditye, 2011	2000, 3000, 4000 ms (+200 ms feedback)	5 min, 24 h, 1 week	Unlimited
	Carbon and Leder, 2006	30 s	80 min	Unlimited
	Carbon et al., 2007	81 s	5 min, 24 h	Unlimited
	Webster and MacLin, 1999	5 min (+8 s top-up)	500 ms	8 s
	Leopold et al., 2001	5,000 ms	150, 300, 600, 1,200, 2,400 ms	200 ms
	Kloth and Schweinberger, 2008	1 min, 24 s (+3.5 s top-up in the first test block)	0–10 min	400 ms
	Leopold et al., 2001	5,000 ms	150, 300, 600, 1,200, 2,400 ms	200 ms
	Leopold et al., 2005	1, 2, 4, 8, 16 s	*nr*	100, 200, 400, 800, 1,600 ms
	Rhodes et al., 2007	1, 2, 4, 8, 16 s	1000 ms	100, 200, 400, 800, 1,600, 3,200 ms
	Rhodes et al., 2003	4,000 ms (+8 s top-up)	500 ms	1,500 ms
	Walther et al., 2013	500 ms	50 ms	300 ms

Priming	Barbot and Kouider, 2012	60, 1,000 ms	0 min	700 ms
	Ellis et al., 1997	5 s	5 min	2.5 s
	Ellis et al., 1993	Unlimited	5 min	Unlimited
	Johnston and Barry, 2006	Unlimited	3–5 min	Unlimited
	Lewis and Ellis, 1999	*nr*	24 h, 7 and 60 days	*nr*
	Maylor, 1998	10 s	4 min, 22 months	10 s
	Rieth and Huber, 2010	17, 50, 150, 400, 2,000 ms	0 min	33, 50, 100 ms
	Walther et al., 2013	500 ms	50 ms	300 ms

*Delay = interval between adaptation and test; feedback = additional adaptor presentation following the adaptation trial and a cover task; top-up = additional adaptation stimulus preceding each test trial; *nr* = not reported.*

### Adaptor/Prime Duration

#### Adaptor Duration

Different presentation durations of the adaptor can cause differences in the strength of the adaptation effects. An increase in the presentation duration of the adaptation stimuli, for example, usually results in a stronger adaptation effect. For relatively short time intervals between adaptation and test phases Leopold et al. (2005) and Rhodes et al. (2007) have investigated adaptation effects on identity alterations (features of identities, who were unknown before starting the adaptation phase, were altered by morphing identities or by increasing or decreasing the identity strength through synthetically altering the person’s features) and adaptation to (initially unknown) distorted faces, by varying the presentation duration of the adaptation stimulus (1,000, 2,000, 4,000, 8,000, 16,000 ms). They demonstrated stronger effects for longer adaptation durations. In fact, this relation can be expressed by a logarithmic function between adaptation duration and effect size. Thus, the adaptation effect constantly increases with longer presentation durations, but the size of the increase progressively decreases. However, it remains unanswered to what extent the approach to the adaptor can proceed. Hence, it is also an open question whether a complete adaptation (meaning a complete adjustment of the mental representation to the perceived face) could be possible with sufficiently long adaptation duration. For long-term adaptation effects (i.e., a break between the adaptation and test phase of 5 min and 24 h) Strobach et al. (2011) found a positive correlation between the presentation duration (comparing durations of 1,000, 2,000, and 3,000 ms) of adaptation stimuli with manipulated eye–mouth distances and the adaptation effect.

#### Prime Duration

For priming, previous studies show rather ambiguous results. Barbot and Kouider (2012), for example, compared a relatively brief duration (60 ms) with a longer duration (1,000 ms) of prime stimulation and found for both durations facilitation in later recognition for initially presented visible face prime stimuli (using familiar faces). However, for stimuli that were invisible to the participants, due to inter-ocular suppression, the authors could demonstrate a facilitated recognition of the primed test stimuli for the short prime duration while the longer prime duration led to a negative priming effect in the test phase, reflecting recognition impairments. Rieth and Huber (2010) systematically investigated priming effects of visible face primes, using face stimuli that were unfamiliar at the beginning of the experiment. By continuously varying the presentation duration of the prime stimuli (17, 50, 150, 400, and 2,000 ms), the authors were able to demonstrate that brief prime durations (up to 50 ms) provoke a facilitation effect while longer prime durations lead to a continuous decrease in performance and again to a negative priming effect (a negative priming effect was reported only for the prime duration of 2,000 ms). Referring to the study of Barbot and Kouider (2012), it might be possible that a negative priming effect would also have been observed for visible familiar face primes if the authors had increased the prime duration.

Study results investigating the priming duration by alternative means, should be taken into account as well. Lewis and Ellis (1999) for example, found a relationship between the number of prime repetitions (using familiar face primes) and the reaction time (to identify a subsequent face), which fits a negative power curve. Thus, the priming effect constantly increases with more prime repetitions (i.e., an increased prime duration), but the size of the increase progressively decreased. Negative priming effects, however, could not be detected in this study. Hence, it can be concluded that prolonged priming in the way of longer presentation durations are qualitatively different from a prolonged priming due to an increase of prime repetitions.

#### Implications of Adaptation and Prime Duration for the Face Space

**FIGURE 2:**
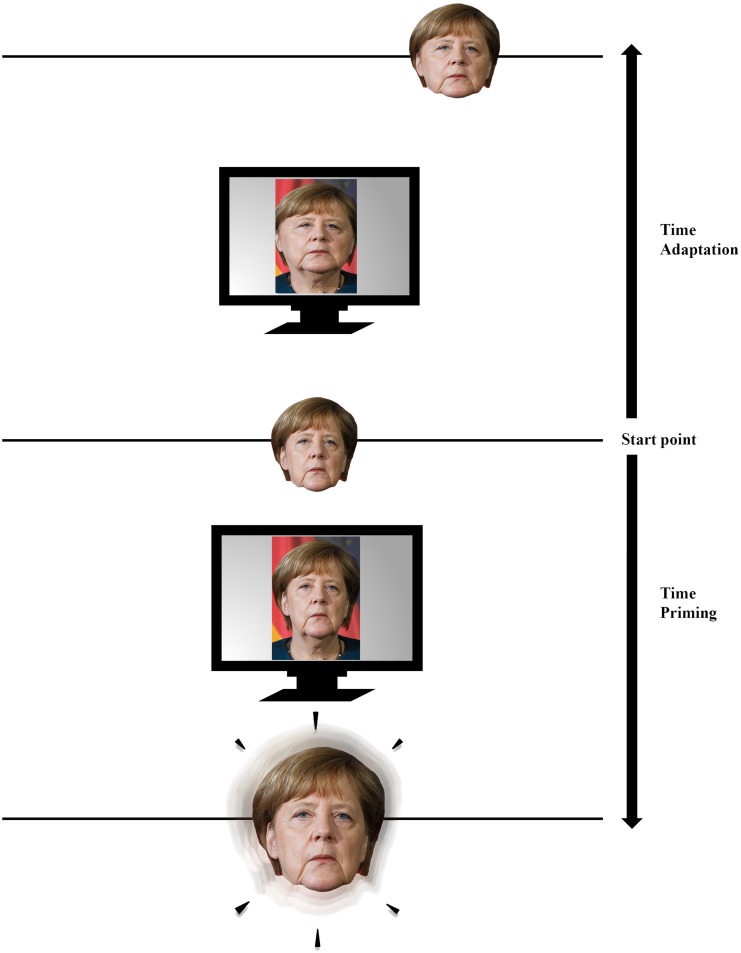
A basic illustration of the processes that occur in face space during adaptation and priming. A picture of German chancellor Angela Merkel is used as an example. Both processes have the same starting point: a mental representation located in face space on a specific face dimension on which a face can vary. During an adaptation process (upwards from center), a strongly manipulated version of the person is then presented on a monitor (in this case a manipulation of the face width was performed). The adaptation to the extreme version subsequently leads to a position shift of the representation toward the adaptor. During a priming process (downwards from center), however, a non-manipulated picture of the person is presented as a prime on a monitor. Although the prime subsequently leads to an activation of the representation within the face space (represented by the “glow” and the enlargement of the image), it does not lead to a position shift. The presented images are used for illustrative purpose only. Permissions and image licenses have been obtained from the copyright holders [Source: © Drop of Light/Shutterstock.com].

Different adaptor and prime durations have very different effects on both phenomena. While a longer presentation duration of the adaptor seems to cause an increasing adaptation effect, a longer presentation duration of a prime leads to a continuous decrease of the effect and can even result in negative priming. In an adaptation process, the representation continuously changes in the direction of the adaptor (Strobach and Carbon, 2013): the longer the presentation duration, the larger the adaptation effect and thus the approximation of the representation to the adaptor. As a result of this approximation, the representation would change its position in face space (along a continuum between the original and the adaptor), in such a way that a greater approximation leads to a larger position shift in face space (Valentine et al., 2016; see Figure 2 for an illustration).

In contrast to adaptation, a positive priming effect does not lead to a shift of the representation within the face space but to an activation increase of the representation. For the greatest possible priming effect, a prime should be presented briefly. This is probably the most effective way to achieve the greatest possible activation of its representation within the face space and a faster processing of the subsequent face stimulus. Rieth and Huber (2010) assumed that a longer prime presentation duration results in a neural habituation and thus leads to a continuous activation reduction of the representation of the previously inspected stimulus. The authors argued that by inhibiting the representation of the previously seen face, a confusion with subsequent faces will be avoided and hence the identification of them will be facilitated. Thus, an accumulation of activations of different representations within the face space does not seem possible.

Furthermore, the reversal into negative priming could be seen as an activation inhibition of face space representations (by habituation of the corresponding neuronal networks; Rieth and Huber, 2010). However, it is open as to whether this negative priming effect could also be explained by an adaptation mechanism and thus a change of the face representation’s position in the face space. The negative priming effect is at least very similar to the effect of a facial adaptation process; both phenomena lead to slower or more difficult recognition of an original face. If one assumes that a mental facial representation never corresponds exactly to what one sees on the presented stimuli (even if the stimuli have not been manipulated), an adaptation process should occur due to the lack of fit between both images (e.g., Clifford et al., 2000; Clifford and Rhodes, 2005). Following this assumption, a longer prime presentation could probably trigger an adaptation process. It therefore remains unclear whether the effect of an extended presentation duration demonstrated by Rieth and Huber (2010) is rather that of a negative priming or that of an adaptation mechanism, or whether both phenomena may be used synonymously in this case.

While longer prime durations lead to a negative priming effect (or adaptation effect) prolonged priming due to an increase of prime repetitions also show a progressive decrease of improvement but no reverse into negative priming (Lewis and Ellis, 1999; Rieth and Huber, 2010). Thus, just like longer prime durations, repeated priming somehow seems to trigger a habituation process as well, leading to a continuous activation reduction of the representation in face space and hence to a decrease in performance. Nevertheless, compared to longer prime durations a high number of repetitions does not lead to negative priming effects. This might be explained by a dishabituation process, caused by interfering sensations (which can be mental or real) in between the prime repetitions. Thus, a previous habituation triggered by a prime could be diminished by the time interval between the prime repetitions, so that further repetitions continue to lead to a positive (although weakened) priming effect. Whether an even higher number of repetitions (even higher than reported by Lewis and Ellis, 1999) could lead to negative priming remains unanswered.

### Test Duration

#### Adaptation Test Duration

The presentation duration of a test stimulus can, similar to the adaptor presentation duration, modulate the magnitude of adaptation effects. An increase of the test duration usually leads to a decrease of the adaptation effect. Using familiarized face stimuli (by implementing a discrimination training before starting the adaptation phase), Leopold et al. (2005) and Rhodes et al. (2007) have investigated facial adaptation effects, varying the presentation duration of the test stimulus (i.e., 100, 200, 400, 800, 1,600, and 3,200 ms). The study results indicate an exponential decay of the adaptation effect, so that the adaptation effect constantly decreases is with longer test stimulus presentation duration, but the size of the decrease is progressively reduced.

#### Priming Test Duration

Presenting face stimuli that were unfamiliar at the beginning of the experiment and by using no time interval between adaptation and test phase, Rieth and Huber (2010) investigated the relationship between test stimulus presentation duration and face priming and were able to demonstrate that longer test durations lead to a decrease of the priming effect (employing test durations of 33, 50, and 100 ms). However, to the best of the authors’ knowledge, there is no systematic investigation of this issue using familiar faces or longer test durations than 100 ms (for results in the adaptation area see, Leopold et al., 2005; Rhodes et al., 2007). This under-representation of studies could be caused by the generally preferred test design. Usually a design is chosen where the test duration depends on the onset of the response the participant is executing, which makes a systematic analysis of the relation between test duration and priming effect difficult. Future research should therefore focus on studies that use a design in which test durations are controlled and determined in advance. In addition, investigations should also include familiar face stimuli, to see if the observed pattern by Rieth and Huber (2010; a decrease of the priming effect due to extended test durations) can also be observed for familiar face stimuli.

#### Implications of Adaptation and Prime Test Duration for the Face Space

It seems that the alteration of the test duration modulates the magnitude of the effects of both phenomena in similar ways. Adaptation studies have shown that an increase in the test duration leads to a decrease in the adaptation effect. In an adaptation process, a longer presentation duration of the test stimulus probably leads to a continuous decrease of the previously generated adaptation effect, due to a readjustment (or re-adaptation) of the representation to the original face (Carbon et al., 2007), since the test stimulus either corresponds to the original face or differs only slightly from the original face (compared to the adaptation stimulus). Referring to the face space this would mean that the adaptation to the adaptor leads to a position shift of the representation within the face space, but a longer test duration reduces this shift by provoking a re-adaptation to the original face and thus a shift of the representation back to the original face space position.

According to Rieth and Huber (2010) not only a prime should be presented briefly, but also the test stimulus, to achieve the greatest possible activation of its representation within the face space and thus the greatest possible priming effect. As mentioned before, a longer prime duration causes a decrease in the priming effect by a decrease of the representation activation due to a neuronal habituation. Because the test stimulus addresses the representation again (regardless of whether it corresponds exactly to the prime stimulus or not), a longer test duration probably has a similar effect as a longer prime duration and leads, as an overstimulation of the representation, to neuronal habituation and thus a decrease in the priming effect as well.

### Delay

#### Delay Within the Adaptation Paradigm

In the early days of face adaptation research, adaptation effects were tested with a time delay of a few seconds or even less between the adaptation and test trials (Webster and MacLin, 1999; Leopold et al., 2001; Rhodes et al., 2003). Such a short delay, however, does not provide much information about the robustness of the effect. Kloth and Schweinberger (2008) conducted one of the first studies to systematically investigate the delay characteristics (using intervals of 1 s up to 539 s) of face adaptation effects with gaze direction information (i.e., the direction the presented identity is looking toward with the eyes while the head is orientated frontally). Although the effects continuously decreased over time, the authors were able to demonstrate those effects on gaze information up to 385 s. Carbon and colleagues extended this research to configural information of the eye–mouth distance and were able to demonstrate (weaker but still significant) adaptation effects even up to a delay of hours and even 1 week (Carbon and Leder, 2006; Carbon et al., 2007; Carbon and Ditye, 2011; Strobach et al., 2011). Thus, face adaptation effects seem to be extremely robust. However, it is still an open issue as to how long exactly an adaptation effect can last and whether these durations vary between different types of facial information (e.g., local information, age, gender, or emotion).

#### Delay Within the Priming Paradigm

The robustness of priming effects in faces is already represented by an adequate number of studies. Face priming effects could be demonstrated in a trial-wise test design for rather short delays (such as milliseconds, seconds, or minutes) between priming and test trials (see, e.g., Ellis et al., 1993, 1997; Johnston and Barry, 2006; Rieth and Huber, 2010; Barbot and Kouider, 2012; Walther et al., 2013) and also for very long delays (such as days, months, or even years) in a design in which both phases were separated (Maylor, 1998; Lewis and Ellis, 1999). Although the face priming effects also become weaker with increasing delays (e.g., Lewis and Ellis, 1999), these effects appear to be similarly robust as face adaptation effects.

#### Implications of the Adaptation and Prime Delay for the Face Space

Both phenomena seem to have a similar robustness and a similar decay pattern of the effects. For adaptation effects, the longest delay between adaptation and test phase investigated so far has been 1 week (Carbon and Ditye, 2011, 2012), which suggests an extremely robust effect. Referring to the face space, these results indicate that changes of the representation are ‘sticky’ and that the shift of representations within the face space is very persistent. However, the continuous decrease of the effect suggests that a readjustment of the representation to the preadaptation status is being performed. Moreover, little is known about the possible reasons for this readjustment to the original representation status before adaptation. It could be that a longer delay between adaptation and test phase provides more opportunities for an exposure (whether mental or real) to the presented identity; because changes of the adaptation stimuli are usually artificial, perceptual experiences outside the experimental setting will mostly contain original faces. This could lead to a re-adaptation process to the original representation in face space, resulting in a continuous reduction of the adaptation effect. Another possible explanation for a re-adaptation could be that to integrate the changes of the adaptation stimuli into the representation permanently, a more frequent and/or longer exposure to the change is necessary (probably in the sense of a threshold that must be overcome). This would indicate that the representations within the face space are extremely stable, so that with adaptation shorter than a potential threshold, a continuous re-adaptation back to the original is performed automatically.

Face priming effects seem to be similar or even more robust than adaptation effects. Studies were still able to demonstrate face priming effects after months or even years (Maylor, 1998; Lewis and Ellis, 1999). Hence, the prime either seems to cause a very stable and long-term activation of the representation located in the face space or it must somehow facilitate a reactivation of it. The latter may appear more likely, since the reactivation of a representation probably consumes fewer cognitive resources and capacities than maintaining an activation over a longer period of time. The continuous decrease of the effect over time is likely to be caused either by a constant decrease of the activation or by a constant decrease of the reactivation capability of the representation located in the face space.

## Transferability

The dimension *transfer* categorizes adaptation and priming effects according to their transferability between different versions of the same image or between different face images and identities. Hence, two approaches can be distinguished: (1) an investigation of the transfer across different changes of specific image dimensions (e.g., orientation, position, or size) or (2) a transfer across different images of the same or different identities (Carbon et al., 2007; Strobach and Carbon, 2013). Studies investigating the transferability of adaptation and priming effects will be discussed according to these approaches.

**FIGURE 3:**
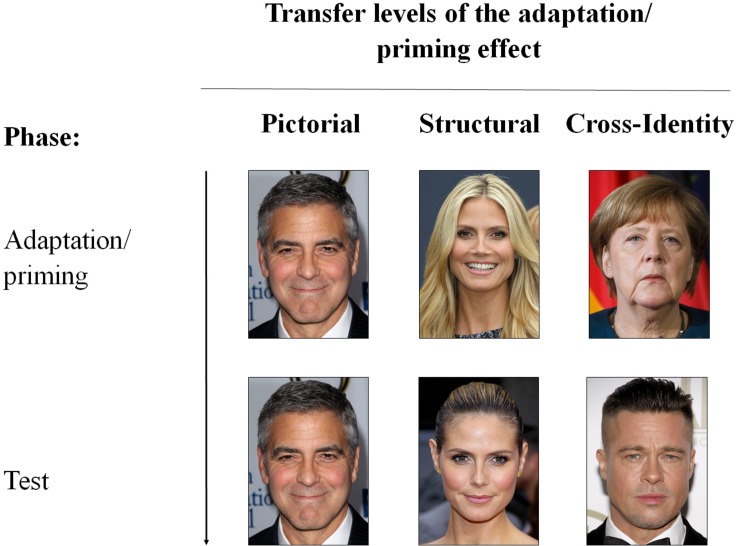
Illustration of the different transfer levels. The presented images are used for illustrative purpose only. Permissions and image licenses have been obtained from the copyright holders [Sources: © Drop of Light/Shutterstock.com, Tinseltown/Shutterstock.com, s_bukley/Shutterstock.com].

The first approach (transferability between images differing in specific image dimensions) is often used to exclude low-level, retinal effects and to understand the role of the altered face dimensions in the storage of faces. This approach is less informative about the identity-specific transfer but rather focuses on image-specific characteristics and their role within the face representations and thus the face space. The second approach compares the identity specificity of the adaptation or priming effects. Here we have three different categories to systemize and compare the transferability (transfer levels). The first category (*pictorial level*) describes an experimental design where the identical image is presented in the adaptation/priming as well as in the test phase. Effects from this category can be contrasted with effects from the second category (*structural level*) in which the adaptation/priming and test image differ from each other but still show the same identity and the third category (*cross-identity level)*, in which different identities are shown in the adaptation/priming and test phase (see Figure 3 for an illustration). By comparing the results of these three categories, it can be determined to what extent adaptation or priming effects are image- or identity-specific. Within the *cross-identity* condition, the strength of divergence between the adaptation/priming and test stimuli can be further varied by investigating the adaptation/priming effects to different groups of individuals, such as gender, ethnicity, age groups, or family members.

In general, the transfer dimension provides important information about the nature of face processing. It reflects the plasticity and flexibility of representations stored in memory, it can reveal common coding principles of faces within different levels of visual processing (from sensory, more retinotopic processing, to a high-level and probably face-specific processing) and can therefore offer important information on the organization of the representations in face space (Webster, 2011; Webster and MacLeod, 2011; Strobach and Carbon, 2013). The different transfer characteristics of all studies reported in this chapter, are summarized in Table 2.

**TABLE 2:** Adaptation dimension or priming task, image transfer dimension and identity transfer dimensions in the selected face adaptation and priming studies reported in this paper.

**Phenomenon**	**Study**	**Adaptation dimension/priming task**	**Image transfer dimensions**	**Identity transfer dimensions**
Adaptation	Afraz and Cavanagh, 2008	Identity	Position	Cross-identity
	Anderson and Wilson, 2005	Identity	Size, viewpoint	Cross-identity
	Barrett and O’Toole, 2009	Gender	*nr*	Cross-identity
	Carbon and Ditye, 2011	Distortion	Position	Pictorial, structural, cross-identity
	Carbon and Ditye, 2012	Distortion	Position	Pictorial, structural, cross-identity
	Carbon and Leder, 2005	Distortion	*nr*	Pictorial, structural
	Carbon et al., 2007	Distortion	*nr*	Pictorial, structural, cross-identity
	Fox and Barton, 2007	Expression	*nr*	Pictorial, structural, cross-identity
	Fox et al., 2008	Identity	Expression	Inter-identity transfer
	Guo et al., 2009	Contrast	Inversion	Pictorial, cross-identity
	Hole, 2011	Identity	Inversion, viewpoint, stretched image	Cross-identity
	Jaquet and Rhodes, 2008	Distortion	Size	Cross-identity
	Jaquet et al., 2008	Distortion	*nr*	Cross-identity
	Jiang et al., 2006	Identity	Viewpoint, face shape, reflectance	Cross-identity
	Kovács et al., 2007	Gender	Position	Cross-identity
	Lai et al., 2012	Age	*nr*	Structural, cross-identity
	Leopold et al., 2001	Identity	Position; size	Cross-identity
	Otten and Banaji, 2012	Expression	*nr*	Structural, cross-identity
	Rhodes et al., 2003	Distortion	Rotation of 45° or 90°	Cross-identity
	Rhodes et al., 2004	Distortion, gender	Size, inversion	Cross-identity
	Strobach et al., 2011	Distortion	*nr*	Pictorial, structural, cross-identity
	Watson and Clifford, 2003	Distortion	Rotation of 45° or 90°, inversion	Pictorial
	Watson and Clifford, 2006	Gender	Rotation of 45° or 90°, inversion, size	Cross-identity
	Webster et al., 2004	Gender, ethnicity, expression	*nr*	Structural, cross-identity
	Yamashita et al., 2005	Distortion	Size, spatial frequency content, contrast, color	Pictorial, cross-identity
	Zhao and Chubb, 2001	Distortion	Size, inversion	Pictorial

Priming	Boehm et al., 2006	Familiarity decision	Inversion	Pictorial
	Bourne et al., 2009	Familiarity decision	Blurring, configural, position	Pictorial
	Brooks et al., 2002	Identification	Inversion, size, position, mirror reversal	Pictorial, structural transfer
	Bruce et al., 1994	Familiarity decision	Color alterations, cartoons	Pictorial
	Bruce and Valentine, 1985	Identification, familiarity decision	*nr*	Pictorial, structural transfer
	Bruce and Valentine, 1986	Familiarity decision	Blurring	Cross-identity
	Ellis et al., 1990	Occupation, familiarity, expression, and gender decision	*nr*	Pictorial, cross-identity
	Ellis et al., 1987	Identification, familiarity decision	*nr*	Pictorial, structural transfer
	Ellis et al., 1993	Identification, expression identification, gender decision	*nr*	Pictorial, structural, cross-identity
	Ellis et al., 1979	Familiarity decision	Trimming to internal or external features	Pictorial
	Goshen-Gottstein and Ganel, 2000	Gender decision	*nr*	Pictorial, structural transfer
	Johnston and Barry, 2006	Occupation and nationality decision	*nr*	Pictorial, cross-identity
	Kaiser et al., 2013	Gender decision	Size	Pictorial, cross-identity
	Rostamirad et al., 2009	Identification	*nr*	Pictorial, cross-identity
	Walther et al., 2013	Identification	Size	Cross-identity
	Young et al., 1994	Identification, familiarity, and gender decision	*nr*	Cross-identity
	Zarate and Sanders, 1999	Gender and ethnicity decision	*nr*	Pictorial, cross-identity

*Adaptation dimension = facial dimension examined within the adaptation paradigm; priming task = identification or classification task within the priming or test phase; image transfer dimension = investigated transferability across specific image dimensions; identity transfer dimensions = investigated transferability according to the three different identity-specific transfer categories; *nr* = not reported.*

### Transfer Between Images Differing in Specific Dimensions

#### Transfer Within the Adaptation Paradigm

Several authors were able to demonstrate adaptation effects regarding the identity or gender of the presented image (features of the identity were altered by morphing the identity with other identities or by increasing or decreasing the identity strength, through synthetically altering the person’s features), despite changes in the (retinal) location of the presented test stimulus (Leopold et al., 2001; Kovács et al., 2007; Afraz and Cavanagh, 2008). The authors either used faces that were unfamiliar (Kovács et al., 2007) when starting the adaptation phase or that were learned in an initial training session (Leopold et al., 2001; Afraz and Cavanagh, 2008). The studies by Kovács et al. (2007) and Afraz and Cavanagh (2008) revealed a reduction of the adaptation effects when changing the position of the test image, suggesting that the effects must include both position-variant and position-specific components.

Furthermore, transfer effects across differences of the (retinal) size between the adapting and test image seem to be possible (at least for faces that were unfamiliar at the beginning of the adaptation phase; Leopold et al., 2001; Zhao and Chubb, 2001; Rhodes et al., 2004; Anderson and Wilson, 2005; Yamashita et al., 2005). However, the study results of Zhao and Chubb (2001), Rhodes et al. (2004), and Yamashita et al. (2005) indicate that the adaptive processes are not insensitive to changes in size but also show some kind of size specificity within the adaptation effect (which is indicated by a reduction of the adaptation effect when changing the size). A similar pattern could also be found for a transfer across different orientations in picture plane. Using faces that were unfamiliar when starting the adaptation phase, Rhodes et al. (2003) and Watson and Clifford (2003, 2006) were able to demonstrate adaptation effects regarding the gender or distortions (e.g., vertically or horizontally, expanded or contracted faces) despite a rotation of the adapting stimuli to either 45 or 90° to the right or the left side. Nevertheless, the study results of Watson and Clifford (2003, 2006) indicate a significantly weaker adaptation effect, when the adaptation and test stimuli were rotated in different directions from the upright position compared to when both were rotated identically. These results suggest again that there must be an invariant component and a dimension-specificity within the adaptation effect.

Previous studies applying an even larger rotation of images by 180° (i.e., an inversion) are very ambivalent in their results. Hole (2011), using familiar face stimuli, and Rhodes et al. (2004), presenting faces that were unfamiliar when starting the adaptation phase, were able to induce similar adaptation effects for upright and inverted faces regarding distortions or the identity of the presented personalities. While Hole (2011) inverted just the adaptation image, Rhodes et al. (2004) demonstrated a transfer of the adaptation effect from (1) inverted adaptation to upright test images and (2) from upright adaptation images to inverted test images. Rhodes et al. (2004), however, used a test design in which those opposite conditions were induced simultaneously (e.g., presenting a contracted, upright face simultaneously with a stretched inverted face in the adaptation phase). Other authors, however, detected an asymmetric pattern. By also presenting faces that were unfamiliar at the beginning of the adaptation phase, they either found much weaker (Watson and Clifford, 2003) or no effects (Watson and Clifford, 2006; Guo et al., 2009) when using inverted adaptation and upright test images, but equally strong effects (compared to no inversion at all) when presenting an upright adaptation and an inverted test stimulus.

Further transfer effects were also observed across alterations of the image relations (e.g., stretching the image vertically) and surface reflectance (i.e., albedo; Jiang et al., 2006; Hole, 2011). Moreover, Yamashita et al. (2005) looked at the transferability of adaptation effects to changes in spatial frequency content, contrast, color and size. While they found strong adaptation effects across changes in contrast, color and size, there was only a weak transferability of the effects to changes in contrast polarity and spatial frequency.

In general, the relation of invariant and variant components of the adaptation effect can vary tremendously for the different images or facial dimensions. For adaptation, alterations on dimensions that are known to affect the recognizability of a face (e.g., contrast and spatial frequency) seem to have a greater influence on the magnitude of the adaptation effect compared to alterations that only marginally affect the recognizability of a face (e.g., size or position; Yamashita et al., 2005; Hole, 2011). However, even for dimensions that affect the recognizability, adaptation effects can still be detected, indicating that although a considerable part of these effects appear to be retinotopic, the effects also indicate an involvement of higher, face-specific processing mechanisms (Hole, 2011). The asymmetric pattern regarding the inversion of the adaptation images observed by Watson and Clifford (2003, 2006) and Guo et al. (2009) might have been obtained due to the use of faces that were initially unfamiliar, since Hole (2011) was able to demonstrate adaptation effects inverting the adaptor by using highly familiar faces. However, it is open as to why Rhodes et al. (2004) were able to prove strong adaptation effects applying an inversion of the adaptor, even though they used faces that were initially unfamiliar. It might be the very different test design the authors used (simultaneously induced opposite adaptation effects) that led to these results.

#### Transfer Within the Priming Paradigm

For priming, Kaiser et al. (2013) and Walther et al. (2013) found effects regarding the recognition of familiar faces, despite differences in the size between the priming and test stimulus. Priming effects could also be generated with a 180-degree rotation (i.e., an inversion) of the prime, using face stimuli with which the participants familiarized themselves in a 1-h training session (Boehm et al., 2006). However, these effects were much weaker compared to the upright prime condition. Similar results could also be observed by Brooks et al. (2002) using familiar face stimuli. They systematically investigated invariances in priming to various metric transformations and found equally strong priming effects when stimuli differed in size, position, and reflectional orientation (mirror reversal). Interestingly, they showed significantly reduced effects when the test stimuli were inverted, pointing to face expert-processing (Schwaninger et al., 2003).

Bruce et al. (1994) were able to demonstrate priming effects from different grayscale images (two levels of gray [black and white] or four levels of gray [black, dark gray, light gray, and white]) on the same colored images (using familiar faces), although these priming effects were much weaker than those that could be detected from one colored image to the next. They also found a similar reduction of priming effects when computer-drawn photographs were presented as the prime and the original photograph as the test stimulus, or vice versa (compared to the condition in which the original images were presented in both phases). By manipulating facial features in the priming phase through blurring, Bourne et al. (2009) were able to show a transfer of identity priming (the task included a popularity rating of the presented famous identities), although here again effects were reduced compared to the unmanipulated condition. Furthermore, identity priming effects seem to be possible even when the test image is trimmed to just the internal features of a familiar (and also initially unfamiliar) face (Ellis et al., 1979).

In general, for priming the pattern seems to be similar to the one seen in the adaptation area: a transfer across manipulations is possible but to some extent the manipulations do affect the magnitude of the priming effect, suggesting invariant and variant components within the priming effect. Whether there is also a similar pattern regarding those dimensions that affect the recognizability of faces more strongly (i.e., dimensions that influence the priming effect more strongly than dimensions that do not have an influence on the recognizability) has not yet been investigated very intensively. The study by Brooks et al. (2002) might suggest a similar pattern to that of adaptation, since an inversion (which is known to impair recognition) generated significantly weaker priming effects than other metric changes (such as size, position, and reflectional orientation).

#### Implications of the Transfer Between Different Image Versions for the Face Space

Altogether, the listed results on adaptation and priming show that the effects are not entirely retinotopic but that there is a component of face-specific processing. The systematic investigation of these face-specific components provides important information about which factors are generally involved in face representations and thus also which dimensions define the face space. Further research could eventually identify and specify the 15 to 22 dimensions of the face space, determined by Lewis (2004) at some point. The above results on adaptation and priming might indicate that factors that qualitatively change faces (and thus affect face recognition, such as contrast polarity) are more likely to be represented in face space than factors that are of a more quantitative or metric nature and thus do not affect recognition, such as size or position.

However, by applying an inversion of a face (which is known to impair recognition and even might lead to non-expert processing of faces, see Schwaninger et al., 2003), very ambiguous results were obtained. Within the adaptation area, Watson and Clifford (2003, 2006) and Guo et al. (2009) observed reduced adaptation effects, by using face stimuli that were unfamiliar at the beginning of the adaptation phase and by inverting the adaptor. Hole (2011), however, also inverted the adaptor but used familiar faces and found adaptation effects were as equally strong as those in upright face stimuli. Thus, an inversion does not seem to affect the perception and processing of familiar faces as much as the perception and processing of initially unfamiliar faces. The facilitated recognition of familiar faces despite an inversion of the face stimulus might be due to a high flexibility (i.e., for example wide-ranging information about the identity) of the representations stored in memory and face space. The high flexibility could allow the identification of a person (and thus also an adaptation of the representation) despite large alterations of the orientation. Unfamiliar faces, on the other hand, are not yet stored in memory as highly flexible representations, so that large alterations of the orientation can be less easily ignored.

However, applying an inversion within the priming paradigm, weaker effects were also observed using familiar faces (Brooks et al., 2002). This may be explained by the differences between the two paradigms priming and adaptation. Within the priming paradigm the presentation duration of the prime is usually much shorter compared to the presentation duration of the adaptor (see section “Implications of the Transfer Between Different Image Versions for the Face Space”). When applying an inversion, the shorter presentation duration could lead to additional recognition difficulties, since more time might be needed to compensate the inversion of the image and to recognize the presented identity and then activate its representation within the face space. Within the adaptation paradigm, on the other hand, the adaptor is usually presented for longer to achieve the greatest possible effect, so that recognition might be easier despite an inversion of the stimuli.

### Transfer on an Identity Level

#### Transfer Within the Adaptation Paradigm

Most studies investigating the transfer of adaptation effects from one image of a familiar identity to a different image of the same identity (*structural level*) were focused on distortions when manipulating the adaptor (e.g., alterations of the eyes–mouth distance; Carbon and Leder, 2005; Carbon et al., 2007; Carbon and Ditye, 2011, 2012; Strobach et al., 2011). All of them were able to find strong transfer effects even though the transfer on the *structural level* often led to a small decrease of the adaptation effect compared to the adaptation effect on a *pictorial level* (Carbon and Leder, 2005; Carbon and Ditye, 2012). Using face stimuli that were unfamiliar at the beginning of the adaptation phase, Fox and Barton (2007) on the other hand, observed equally robust adaptation effects on the *pictorial* and *structural level*. However, they did not distort the presented stimuli but investigated adaptation effects to different emotional expressions.

The same authors were also able to transfer the demonstrated effects across different identities (*cross-identity level)*, irrespective of the gender of the presented identities (Fox and Barton, 2007). Although these adaptation effects on a *cross-identity level* were significant, they were weaker compared to the effects on the *pictorial* or *structural level*, suggesting that the generated effects are at least partially identity-specific. Fox et al. (2008) reversed this paradigm by investigating adaptation effects on identity alterations and their modulation by congruency of facial expressions (using familiar as well as initially unfamiliar face stimuli). Interestingly, they did not find any impact of expression congruency on the identity effects, which indicates that identity perception is independent of emotional expressions (Fox et al., 2008). However, other authors who also investigated adaptation effects on different emotional expressions found limitations in the transferability of the effects. Using faces that were unfamiliar when starting the adaptation phase, Otten and Banaji (2012) reported a robust transferability of expression effects across images of the same identity and across images of a different identity but significantly weaker effects for images of individuals of a different ethnicity.

For configurally distorted famous faces, several studies were able to demonstrate a robust transfer to other identities, even though the magnitude of the effects decreased compared to the *pictorial* or *structural level* (Carbon et al., 2007; Carbon and Ditye, 2011, 2012; Strobach et al., 2011). However, also for configurally distorted (initially unfamiliar) faces, a limited transferability to other ethnicities could be observed (Jaquet et al., 2008), indicating an ethnic specific component within the effect. Additionally, Jaquet and Rhodes (2008) investigated the transferability of adaptation effects on configural alterations (presenting face stimuli that were initially unfamiliar) across gender and found a partial transfer (i.e., a weaker but still significant effect for the different-gender condition than for the same-gender condition), suggesting both common and gender-specific components within the adaptation effect. A gender adaptation effect (using as well-initially unfamiliar faces), on the other hand, was largely transferable across different identities and thus did not seem to have an identity-specific component (Webster et al., 2004). Moreover, the study also revealed differences in category boundaries selected in the preadaptation assessment (observers categorized images of a gender-morph-continuum to estimate the category boundary of male and female), indicating that observers tended to select a category boundary that was shifted to their own gender. These differences in category boundary were also observed with different ethnicities (Asian and Caucasian), leading to a shift of the observer’s boundary toward their own ethnicity. However, this shift was not as intense when observers had already been exposed to the other race for a longer time (e.g., Asians living in the US for at least 1 year). Additionally, other authors have found an impaired identification of the face’s gender in other race faces (O’Toole et al., 1996).

A transfer of adaptation effects between other social groups was found by Barrett and O’Toole (2009). They investigated adaptation effects (using face stimuli that were unfamiliar at the beginning of the experiment) on gender and demonstrated a transfer of these effects across different age groups (Barrett and O’Toole, 2009). However, by presenting familiar face stimuli, Lai et al. (2012) investigated adaptation effects on age differences and observed that they were just partly transferable across different identities, suggesting both identity-variant and identity-specific components within the adaptation effect.

#### Transfer Within the Priming Paradigm

Face priming effects seem to be equally transferable to different images of the same identity (*structural level*) as adaptation effects (Bruce and Valentine, 1985; Ellis et al., 1987, 1993), although the priming effects seem to decrease the more dissimilar the prime and the test images are. Ellis et al. (1987) systematically investigated the transferability of priming (using famous faces) by applying a three-level differentiation regarding the similarity of the presented test stimuli to the prime: identical, similar, and dissimilar (the similarity of the stimuli was rated in advance by an independent jury). The priming effect seemed to be greatest when the prime and the test image were identical, less when they were similar, and least when they were dissimilar (but still representing the same person).

Several studies investigating priming effects on a *cross-identity level* focused on associate priming and thus compared highly associated couples (e.g., Siegfried and Roy) with less associated identities (e.g., Angela Merkel and George Clooney). In these studies, Bruce and Valentine (1986) and Young et al. (1994) observed strong priming effects on famous highly associated identities. While a transfer of the priming effect to a highly associated identity seems to be successful, a transfer to other ethnically related identities apparently does not work. By using face primes that were unfamiliar at the beginning of the experiment, Rostamirad et al. (2009) demonstrated a facilitation effect in recognition when the test face was preceded by the same priming face, an inhibition effect when it was a different face and an even greater inhibition when the face belonged to the same ethnic group as the prime. Zarate and Sanders (1999) also investigated priming effects (using faces that were initially unfamiliar) across different ethnicities and gender. They could not find a relation between the strength of the priming effect and ethnic and gender similarity of the prime and test image, but observed that the magnitude of the priming effect was rather modulated by the general similarity of the stimuli. The authors suggest that the facilitation effect is not generated through the activation of a superordinate abstract concept (e.g., gender or ethnicity), but that the priming effect is rather prime-specific and depends on the inter-item similarity.

In line with these results, Ellis et al. (1990) found that, by systematically investigating the transferability of priming effects (using familiar and initially unfamiliar face stimuli) to different categorization tasks (familiarity, occupation, gender, and expression tasks), priming effects only occur for decisions in the test phase that require an identification of a face and not a classification according to a higher concept. Thus, a priming effect was observed for familiarity and occupation tasks in the test phase but not for gender or expression tasks. On the other hand, any encounter with a face seems to be sufficient to cause priming (meaning that the magnitude of a priming effect does not depend on the task given in the priming phase, but obviously does depend on the task given in the test phase). Nevertheless, the study results of Johnston and Barry (2006) do not seem to fully fit into this picture. As in the study by Ellis et al. (1990), the authors found repetition priming effects for an occupation decision task. However, by using familiar faces, Johnston and Barry (2006) were also able to demonstrate a priming effect for a nationality decision task in the test phase, which, according to the Ellis et al. (1990) argumentation (that only tasks that require an identification of the face show priming effects), should actually not exist. Other authors also disagreed with the argumentation of Ellis and colleagues, since they were able to find priming effects (using face stimuli that were unfamiliar at the beginning of the experiment) for a gender decision task (Goshen-Gottstein and Ganel, 2000). However, Ellis et al. (1990), Goshen-Gottstein and Ganel (2000), and Johnston and Barry (2006) investigated priming effects across different categorization tasks only on a *pictorial level*. They did not consider the transferability of the effects on a *structural* or *cross-identity level*. Hence, in their test design higher concepts (such as gender) could only be addressed by the categorization of the stimuli (in, e.g., female or male). However, since a systematic comparison of the categories on a *cross-identity level* [i.e., comparing the transferability of priming effects of an identity of one category (e.g., female) to either a different identity of the same category or an identity of the opposite category (e.g., male)] was not performed, the results give little assurance as to whether higher concepts were really primed or not.

#### Implications of Identity Transfer for the Face Space

Both phenomena, adaptation and priming, seem to transfer quite well across different images of the same identity and thus provide further evidence that adaptation as well as priming effects do have an identity-specific component. Furthermore, the results regarding the transferability on a *structural level* suggest a high flexibility of the representations stored in memory and face space, since they seem to be altered and activated despite significant alterations of the images presented in the adaptation and priming paradigms. Thus, they must somehow contain either wide-ranging information about the identity or stable, basic and minimalistic face structures that allow an identification of a person despite large and diverse changes. The basis on which the recognition of a person’s face occurs is not clear yet. However, the fact that adaptation effects can be generated through altering very diverse information might indicate that the representations rather contain a wide range of different face information than just a very basic structure.

Regarding transferability on a *cross-identity level*, the reported findings on adaptation provide clear evidence that adaptation effects are transferable to different identities. This suggests a hierarchical processing of faces, where adaptation also affects superordinate face categories, indicating a distinction of different social groups (e.g., different ethnicities, gender, etc.) in facial processing. Referring to the face space, this would mean that adaptation not only alters the representation of the presented identity but also the representation of the social group(s) to which the person belongs. This suggests that either different modules or sub-face spaces must exist (that probably show some overlap between each other) or different prototypes for each social group, which can be altered through adaptation. Thus, adaptation would alter the common underlying face structures and lead to an alteration not just of that identity presented in the adaptation paradigm but also of all other faces located close to the identity or within the same social group. The reported results regarding the category boundary further suggest that there are stronger adaptation effects to the sub-face spaces individuals encounter most and also that these more familiar sub-face spaces are more differentiated.

Furthermore, there seem to be differences in the transferability of adaptation effects depending on the type of adaptation information. Face information that is more transient or more variable (such as facial expression or age) appears to transfer more easily (see the reported results of, Fox and Barton, 2007; Fox et al., 2008; Barrett and O’Toole, 2009; Lai et al., 2012) than information that is invariant (such as configural face information, gender, or ethnicity), suggesting that this information type has a more subordinate role in facial perception and processing and thus also in the face space. However, on a *cross-identity* level (i.e., a transfer to other identities) more variable information also shows slight limitations of the transfer to other identities (Fox and Barton, 2007; Lai et al., 2012), indicating that it cannot be neglected completely when investigating face-specific adaptation effects. Thus, it does have a face-specific component within the adaptation effect and therefore must also be somehow represented in face space. However, the face-specific component of the effect seems to be somehow smaller compared to the level of face specificity within the adaptation effects obtained with more invariant information. This indicates that the representation of more variable information in face space must also be somehow weaker compared to the representation of more invariant information.

For priming, the picture regarding the transferability on a *cross-identity level* seems to be more ambiguous than for adaptation. Many of the reported results indicate transferability to different identities but this transfer rather depends on the similarity of the presented images than on belonging to the same or a different superordinate social group. Thus, referring to the face space, an activation of a representation of an identity might not automatically lead to an activation of a higher-level prototype or a sub-space. The results rather indicate that, within the face space, just those identities that are located very close to the primed identity (due to their similarity on many face dimensions) might also be affected through priming. However, the outcomes regarding the transferability of priming effects to associated individuals suggest that an activation of representations that are more distant to the primed identity within the face space is also possible. Thus, the activation of a representation might automatically lead to an activation of the representation of the associated person, due to the strong associative and exclusive bond between them. Another explanation could be that by activating a representation, a higher concept under which both associated persons are to be classified is activated as well. As a result, not only the representations of the associated persons would be activated, but also representations of possible other persons belonging to this concept. Although this idea would clearly contradict the results of Zarate and Sanders (1999; demonstrating that the priming effects rather depend on the inter-item similarity than on the belonging to the same concept), it would support the results of Goshen-Gottstein and Ganel (2000) and Johnston and Barry (2006), who demonstrated priming effects for nationality and gender tasks. However, the results of these studies should be taken into account with caution, since it is not clear yet whether the implemented categorization tasks can really address higher concepts.

## Discussion

Adaptation and priming are two established paradigms in the face literature. Both paradigms and their associated phenomena can assist a better understanding of face recognition and face representation. Regarding the face space framework, up to now the literature has mainly focused on face adaptation while largely neglecting face priming. By reviewing and comparing the literature on face adaptation and face priming within one paper, this work aims to create an overall picture of both phenomena and their contributions to the face space. While face adaptation is a phenomenon that seems to alter a representation, leading to a shift of the representation within the face space, face priming mainly activates such a representation. The reported studies show that these effects can be influenced by modifications of the temporal components of adaptation and priming.

### Summary of the Temporal Characteristics of Adaptation and Priming and the Consequences for the Face Space

A longer adaptation duration causes an increasing adaptation effect (Rhodes et al., 2007; Strobach et al., 2011) and therefore a larger position shift of the representation within the face space. A longer prime duration, however, leads to a decrease of the priming effect (Rieth and Huber, 2010) and therefore to an activation reduction of the facial representation within the face space. This activation reduction might be an automatized mechanism to avoid a confusion with other faces presented afterward. However, a longer prime duration can sometimes even cause a negative priming effect (Rieth and Huber, 2010; Barbot and Kouider, 2012). Since negative priming and adaptation seem to be identical regarding their behavioral outcome (they both lead to a slower or more difficult recognition), the negative priming effect could also be that of an adaptation process (evoked due to the lack of fit between the presented facial stimulus and the mental representation). Thus, a longer prime duration might function as an adaptor and might lead to a shift of the facial representation within the face space toward the prime.

Compared to the adaptation duration, a longer presentation of the adaptation test stimulus does not cause a greater adaptation effect but rather decreases it Leopold et al. (2005) and Rhodes et al. (2007), since it leads to a re-adaptation process and thus to a shift of the facial representation within the face space back to the original position. A longer presentation of the prime test stimulus also causes a decrease of the priming effect (Rieth and Huber, 2010), probably because the test stimulus addresses, just like the prime, the mental face space representation and thus leads, as an overstimulation of the representation, to a neuronal habituation and activation reduction.

The effects of both phenomena, however, can be equally robust, since they can last up to a week or (at least priming effects) up to several months (Maylor, 1998; Lewis and Ellis, 1999; Carbon and Ditye, 2011, 2012). Thus, an adaptation can probably lead to a very persistent shift of the mental representation within the face space, whereas priming causes a long-lasting activation or a facilitated reactivation of the facial representation. However, the effects of both phenomena decrease, the more time elapses between the adaptation/priming and the test phase. Within the context of adaptation, a possible explanation for the continuous decrease of the effect might be an increased possibility of exposure to the presented identity (which possibly causes a re-adaptation back to the original representation and thus decreases the effect), during a long delay. Another possible explanation could be that the re-adaptation process is an automatic process due to the robustness of the original facial representation (which presumably is built up over a longer period of time) and which does not integrate transient alterations permanently unless they are presented for longer and/or more frequently. The continuous decrease of priming effects over time, however, might be caused by either a constant decrease of the activation of the representation or a decrease of the reactivation capability of the representation located in face space. Since a longer priming duration seems to lead to an activation reduction to inhibit a confusion with other faces seen subsequently, a long-term maintenance of activation does not seem plausible (also for shorter priming durations). A facilitated reactivation, however, would probably not inhibit the recognition of subsequent faces (since it would only lead to a full reactivation of the representation, if the identity is presented again) and thus might be more likely.

### Summary of the Transfer Characteristics of Adaptation and Priming and the Consequences for the Face Space

Not only the temporal components of adaptation and priming give us information about the facial perception, the storage and thus the face space, but also the transferability of adaptation and priming effects contribute to the understanding of it. The listed study results reveal that a transfer of adaptation and priming effects across alterations of specific image dimensions is possible, but to some extent the alterations do affect the magnitude of the effects (meaning that the effects usually decrease when they are transferred; see, e.g., Bruce et al., 1994; Brooks et al., 2002; Yamashita et al., 2005; Kovács et al., 2007). This indicates that although a considerable part of the effects seems to be retinotopic, there does exist some kind of face-specific component too. Nevertheless, the results also show that dimensions that qualitatively change faces and thus affect their recognition (e.g., contrast polarity or spatial frequency) have a greater impact on the adaptation and priming effects than dimensions that are of a more quantitative or metric nature (e.g., size or position) and do not affect the recognition as much (see, e.g., Brooks et al., 2002; Yamashita et al., 2005). Thus, the dimensions that have a greater impact show a greater face specificity and are therefore more likely to be represented in face space than dimensions that do not affect the adaptation and priming effects as much.

Adaptation and priming effects can be observed not only despite changes in specific image dimensions, but also despite a presentation of a completely different image of the identity in the test phase (Bruce and Valentine, 1985; Ellis et al., 1987; Carbon and Leder, 2005; Carbon et al., 2007). Thus, the representations within the face space must somehow be very flexible. They must either contain wide-ranging information about the identity or stable but minimalistic face structures that allow an identification of an identity despite large and diverse changes. Since adaptation effects can be evoked by altering very diverse face information, the latter might be more likely.

Adaptation and priming effects regarding the transferability on a *cross-identity level* reveal that both kinds of effects are (at least partly) transferable to different identities (Bruce and Valentine, 1986; Carbon et al., 2007; Strobach et al., 2011). The reported findings on adaptation indicate a hierarchical processing of faces, where adaptation not only alters the representation of the presented identity but also affects superordinate concepts (see, e.g., Jaquet and Rhodes, 2008; Jaquet et al., 2008). This suggests that there must exist different sub-face spaces or different prototypes for each superordinate concept within the face space. Thus, by altering the representation of a specific identity, underlying face structures of superordinate concepts would be altered toward the adaptor too. The findings further suggest that there are stronger adaptation effects for the sub-face spaces individuals encounter most and that these sub-face spaces are more differentiated than more unfamiliar ones (O’Toole et al., 1996; Webster et al., 2004). The listed results on priming, on the other hand, are ambiguous. Some studies indicate that the transferability of priming effects to other identities rather depends on the similarity of the presented images than on the belonging to (and thus activation of) a common sub-face space (Zarate and Sanders, 1999). Other study results, however, suggest, that priming is able to activate representations of identities that are strongly associated with the primed identity (e.g., Young et al., 1994). However, it is not clear yet whether this activation of other representations occurs due to an activation of a higher sub-face space under which the associated persons are to be classified or due to an associative and exclusive bond between these identities.

### Future Adaptation and Priming Studies and the Face Space

The two phenomena adaptation and priming demonstrate that perceived faces can change our facial perception and storage and hence our face space significantly. Adaptation and priming studies manipulating the temporal factors help identify the best temporal structure of both paradigms (in the way of generating the greatest possible effects; e.g., for adaptation a long adaptor and a short test stimulus, for priming both: a short prime and a short test stimulus). A systematic manipulation of temporal parameters may also provide information about the underlying mechanisms of the two phenomena. It seems that the presentation of a stimulus could initially lead to a priming effect. However, prolonging the presentation of the stimulus might possibly induce an adaptation effect (see the results on negative priming). Thus, it could be assumed, that it depends on the presentation duration of the adaptor/prime, which phenomenon occurs.

However, the study by Walther et al. (2013) indicates that the ambiguity of the test stimulus should rather be considered as the decisive factor. The authors were able to demonstrate both phenomena within one paradigm. While adaptation effects were induced by presenting very ambiguous test stimuli, unambiguous test stimuli provoked priming effects. Thus, it still seems unclear which specific parameters (e.g., temporal parameters or the ambiguity of the test stimuli) are crucial for the occurrence of one or the other phenomenon. Future studies should therefore focus more on the investigation of specific parameters using combined (adaption and priming) paradigms to better understand and distinguish both phenomena.

Furthermore, studies on temporal characteristics provide important information about the robustness of the adaptation and priming effects and the hereby-created long-term transformation of the face space (i.e., persistent shifts of representations and sub-face spaces, a permanent facilitation of activation as well as the pattern of decay of those alterations). However, it is still unclear which mechanisms are causing the continuous decrease of the effects over time, at least for adaptation. Future studies should therefore specifically investigate the two possible mechanisms responsible for the re-adaptation (i.e., increased exposure to the original image of the presented identity or automatic re-adaptation process due to the robustness of the original facial representation).

The investigations on the image dimensions of adaptation and priming stimuli reveal that some face information is more important and thus probably more likely represented in the face space than other facial characteristics (e.g., invariant information, such as gender, ethnicity, etc. is more likely included in face space than variant information, such as size or location). However, due to a lack of systematic studies on this topic, the dimensions on which all faces vary within the face space [according to Lewis (2004) there should be from 15 up to 22 dimensions] could not yet be determined. Nevertheless, the view that only an exclusive number of dimensions exist in face space and thus facial information other than those dimensions is not considered in facial perception, should be questioned. The reported studies show that facial information that seems to be less important for facial perception (since it has a weaker impact on adaptation and priming effects) still affects the perception of faces and thus also the face space. Perhaps this categorical view of the face space dimensions should be reconsidered and it should be further investigated whether the very diverse facial information is just represented with a different weighting within the face space. Future research should therefore systematically compare adaptation and priming effects on variant and invariant facial information.

The reported studies investigating the transferability of effects further show that the facial representations located in face space are very flexible (i.e., a recognition is possible despite large alterations of the presented face) and that there must be some kind of sub-face spaces representing higher concepts. While adaptation clearly has an effect on these sub-face spaces, no final conclusion can be drawn yet as to whether priming also activates higher concepts. Thus, future studies should focus on a systematic investigation of priming effects on different superordinate concepts to further clarify this topic.

Finally, it can be stated that priming probably can add as much to the understanding of face space as adaptation. It would therefore be recommendable to consider priming more closely in this still challenging field of face research in the future.

## RÉSumÉ

Face adaptation and priming are two phenomena that seem to differ tremendously. While adaptation leads to a shift of representations within the face space, priming rather activates these representations without shifting them (see Figure 2). However, both paradigms alter subsequent face recognition and thus, can be used to gain a better understanding of face recognition, representation and hence the face space. By analyzing the characteristics of the two different effects, both phenomena can give us detailed information about the content, the structure and the flexibility of the face space. The adaptability or priming of specific face information, for example, provide insight into what facial dimensions are stored in face space. The comparison of different facial information may also reveal a different weighting of the information stored in face space (e.g., invariant information seems to be more relevant than variant information). The transferability of effects can reveal information about the structure of the face space (e.g., division into sub-face spaces). The robustness and decay of effects, however, give insight into how flexible or stable representations are within the face space. Furthermore, the specific temporal characteristics of the two paradigms (e.g., adaptor/prime duration) might reveal information about the underlying mechanism of both phenomena. Thus the presentation of a stimulus may initially lead to a priming effect (i.e., a pure activation of the representation). However, if the stimulus is presented for a longer time, an adaptation effect might be induced. The systematic evaluation of the adaptation and priming literature presented here, highlights the valuableness of both paradigms for the investigation of face recognition and representation. It also reveals that priming (although often neglected by face-space literature) is an equally useful tool as adaptation to explore the face space.

## Author Contributions

C-CC and TS conceived of the conceptual idea. RM wrote the manuscript with input from all coauthors (C-CC, SU, and TS). TS supervised the process of writing. All authors provided critical feedback and helped in shaping the manuscript.

## Conflict of Interest

The authors declare that the research was conducted in the absence of any commercial or financial relationships that could be construed as a potential conflict of interest.
